# Rotating nasal masks with nasal prongs reduces the incidence of moderate to severe nasal injury in preterm infants supported by noninvasive ventilation

**DOI:** 10.5935/0103-507X.20220022-en

**Published:** 2022

**Authors:** Paulo André Freire Magalhães, Ana Carolina Gusmão D’Amorim, Elis Fernanda Araújo Lima de Oliveira, Maria Evelyne Albuquerque Ramos, Ana Patrícia Duarte de Aquino Mendes, Juliana Fernandes de Souza Barbosa, Cyda Maria Albuquerque Reinaux

**Affiliations:** 1 Postgraduate Program in Rehabilitation and Functional Performance, Research Group of Neonatal and Pediatric Physical Therapy, Universidade de Pernambuco - Petrolina (PE), Brazil.; 2 Real Hospital Português de Beneficência em Pernambuco - Recife (PE), Brazil.; 3 Department of Physical Therapy, Universidade Federal de Pernambuco - Recife (PE), Brazil.

**Keywords:** Masks, Noninvasive ventilation, Protective factors, Nasal septum, Nose/injuries, Soft tissue injuries, Infant, premature

## Abstract

**Objective:**

To investigate the association between noninvasive ventilation delivery devices and the incidence of nasal septum injury in preterm infants.

**Methods:**

This retrospective singlecenter cohort study included preterm infants supported by noninvasive ventilation. The incidence of nasal injury was compared among three groups according to the noninvasive ventilation delivery device (G1 - nasal mask; G2 - binasal prongs; and G3, rotation of nasal mask with prongs). Nasal injury was classified according to the National Pressure Ulcer Advisory Panel as stages 1 - 4. Multivariate regression analyses were performed to estimate relative risks to identify possible predictors associated with medical device-related injuries.

**Results:**

Among the 300 infants included in the study, the incidence of medical device-related injuries in the rotating group was significantly lower than that in the continuous mask or prong groups (n = 68; 40.48%; p value < 0.01).

The basal prong group presented more stage 2 injuries (n = 15; 55.56%; p < 0.01). Staying ≥ 7 days in noninvasive ventilation was associated with a higher frequency of medical device-related injuries, regardless of device (63.81%; p < 0.01). Daily increments in noninvasive ventilation increased the risk for nasal injury by 4% (95%CI 1.02 - 1.06; p < 0.01). Higher birth weight indicated protection against medical device-related injuries. Each gained gram represented a decrease of 1% in the risk of developing nasal septum injury (RR: 0.99; 95%CI 0.99 - 0.99; p < 0.04).

**Conclusion:**

Rotating nasal masks with nasal prongs reduces the incidence of moderate to severe nasal injury in comparison with single devices. The addition of days using noninvasive ventilation seems to contribute to medical device-related injuries, and higher birth weight is a protective factor.

## INTRODUCTION

Preterm birth stands out as the main risk factor for respiratory failure due to incomplete anatomical differentiation and an inadequate amount of pulmonary surfactant, leading to diffuse alveolar atelectasis, edema and cell injury.^(1-3)^ These conditions justify the need for ventilatory support and admission to the neonatal intensive care unit (ICU).^(1-5)^

Greater survival of preterm infants with progressively smaller birth weight and gestational age has been widely reported in the literature worldwide. Nevertheless, this fact increases the risk of future morbidities (e.g., motor, cognitive, and sensory disabilities) and is a reason for attention and study by health professionals.^(1,6)^ Moreover, invasive mechanical ventilation (IMV) contributes to harmful effects such as nosocomial infections, pressure injury, and ventilator-induced diaphragmatic dysfunction, thus delaying extubation and increasing mortality.^(7)^

As an alternative to IMV, noninvasive ventilation (NIV) [continuous positive airway pressure - CPAP or NIPPV - nasal intermittent positive airway pressure] has been widely used with efficiency in neonates, reducing adverse events. Noninvasive ventilation is used through different delivery devices, such as nasal prongs and/or masks, which are the most used in health services due to their easy application and lower cost. ^(8-10)^

The continuous use of NIV, although beneficial, can cause complications, including lesions in the nasal septum and columella.^(11-21)^ These injuries are frequent, as prolonged pressure leads to impaired tissue perfusion with resulting pressure injury, since premature infants have cutaneous vulnerability and specific anatomical factors related to gestational age, such as final vascularization of the columella and nostrils, which favors the appearance of lesions.^(21)^

Nasal septum injury has been responsible for 6% of adverse events in the neonatal ICU,^(18)^ and its prevalence has increased 20 - 100% in preterm infants receiving NIV, regardless of the device used and multidisciplinary care, which provides a clear opportunity to improve skin care outcomes.^(21)^ Thus, resources and strategies to prevent the incidence of nasal septum injury should also be considered. In this sense, various NIV delivery devices are available, and few studies have investigated the incidence, tolerance and differences between nasal pressure injury in infants managed by NIV.(11-18,20,21) Of note, only two studies(11,18) evaluated the incidence and severity of nasal injury when rotating two different nasal devices (masks and prongs). These studies reported diverging results regarding the occurrence of injury in association with the rotation NIV delivery device.

This retrospective cohort study aimed to investigate the association between NIV delivery device protocols and the severity of nasal septum injury and to determine the relative risks of factors associated with the incidence of nasal injury and between the stages of nasal septum injury in preterm infants in the neonatal ICU.

## METHODS

This is a retrospective cohort study of open therapeutic intervention in which data were collected from electronic medical records at a neonatal ICU located in northeastern Brazil from 2015 to 2020. This work was approved by Ethics Committee.

The obtained sample was nonprobabilistic due to the availability of the NIV delivery device type (mask, prong or both), and composed of infants of both sexes, less than 37 weeks gestational age, and admitted consecutively to the NIV in the neonatal ICU. Exclusion criteria were receiving NIV treatment less than 24 hours, the presence of preexisting nasal injuries and/ or deformities, infants transferred from other hospitals to our neonatal ICU who received NIV over 24 hours, shock and coagulation disorders or who displayed nasal injury on the occasion of the physical examination upon admission, intubated at admission to the neonatal ICU or records with incomplete data.

Preterm infants were designated according to the consecutive admissions to the NIV in the neonatal ICU, and the protocol was instituted from the acquisition period of the NIV delivery devices in the service. Each device was not introduced at the same time; as a single device protocol was initially implemented with nasal prongs, next, nasal mask insertion was requested, and finally, the rotation protocol with prongs and nasal masks was instituted.

**Figure 1:**
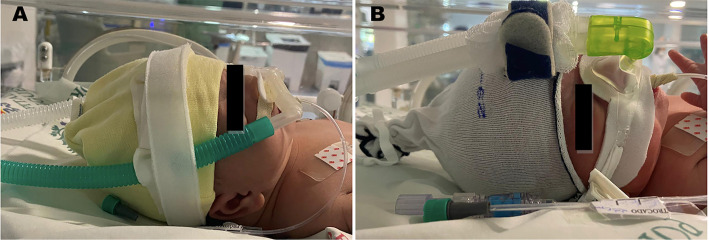
Illustrative image of infants using (A) binasal prongs and (B) nasal masks during noninvasive ventilation therapy in our neonatal intensive care unit.

**Figure 2:**
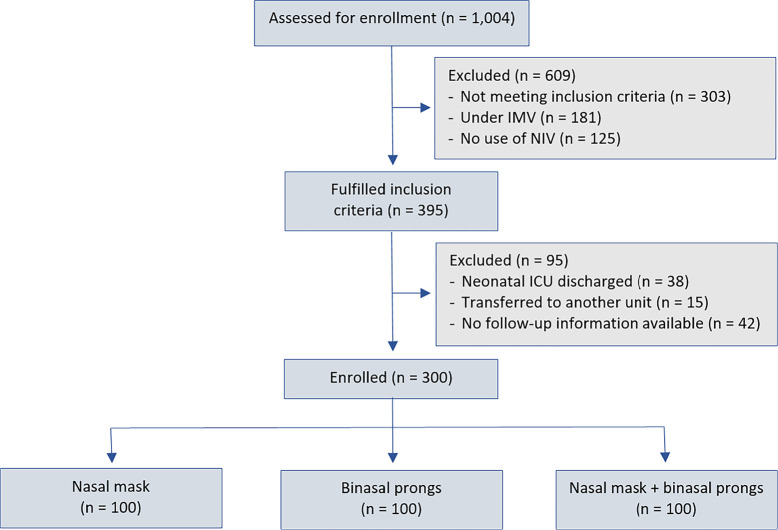
Patient recruitment and selection process. IMV - invasive mechanical ventilation; NIV - noninvasive mechanical ventilation; ICU - intensive care unit.

Participants were divided into three groups over time as devices were acquired in the neonatal ICU: Group 1, first 100 infants receiving NIV by short binasal prongs (Figure 1A); Group 2, first 100 infants receiving NIV by nasal mask (Figure 1B); and Group 3, first 100 infants receiving NIV by rotation of nasal prongs and nasal masks every 12 hours. Figure 2 describes a flowchart of the patient recruitment and selection process. All newborns were managed with the same type of ventilator (Servo I, Maquet, Sweden) for NIV delivery during the study period using short binasal prongs (Hudson short binasal prongs) and/or masks (Dräger BabyFlow® mask) with appropriate and recommended sizes as per the manufacturer’s instructions.

Data were collected by an active search of electronic medical records through two structured forms elaborated by the authors. The first form addresses neonatal and treatment-related variables (date of birth, gestational age, sex, weight, length of NIV, NIV modality, and type of NIV delivery device).

The second refers to the assessment of pressure injury usually caused by the use of medical devices (medical device-related injuries - MDRIs), such as respiratory devices (prongs, masks).^(22)^ Medical device-related injuries were classified according to the National Pressure Ulcer Advisory Panel (NPUAP) numerically classified as stage 1 or 2 or 3 or 4, based on the deepest tissue type exposed: stage 1 pressure injury: nonblanchable erythema of intact skin; stage 2 pressure injury: partial-thickness skin loss with exposed dermis; stage 3 pressure injury: full-thickness skin loss; and stage 4 pressure injury: full-thickness skin and tissue loss.^(13)^ The staging system also included deep tissue injury and unstageable pressure injuries, where the depth of tissue injury was not known.

Medical device-related injury documentation in the electronic medical records was performed by the physiotherapy team after visual inspection of the newborns through reference images. One single researcher trained/skilled in the use of the MDRI staging assessment tool^(13)^ retrospectively reviewed and classified MDRI images in the patient health records.

Managing MDRIs related to NIV is part of the collaborative practice between nurses and physical therapists in the routine of our neonatal ICU. The neonatal ICU nurses actively participated in patient quality of care and safety on preventing MDRI through the choice/ application of skin barrier and management of the nasal injury, while the physiotherapists were responsible for respiratory care, including handling NIV delivery devices and classifying MDRI.

All groups received the same general care protocol for handling the NIV delivery device for preventing MDRI during NIV therapy by trained physiotherapists and nurses with standard operating procedures as follows: a) daily verification of appropriately sized Dräger BabyFlow® mask and Hudson prongs as per the manufacturer’s instructions was used for providing NIV; b) skin barrier (DuoDERM Extra Thin Dressing) was applied at the pressure points; c) massage with two fingers gently rubbing the alar cartilage area before nasal aspiration was used to facilitate secretion shear and then assist in its removal around the middle turbinate; d) nasal aspiration with tube caliber appropriate to the infant’s weight (when necessary); e) the gas delivered in both the NIV delivery devices was heated and humidified to attain a gas temperature of 37°C at the level of the nostrils. The humidifier used in both groups had a flow-based Servo-humidification control mechanism to ensure appropriate humidification; f) monitoring for position of nasal prongs/mask, prongs/mask displacements and fixation of tubes to cap; g) positioning of infants with nest cotton cushion to provide support and posture to the movements, optimizing motor development and promoting calmness in the behavioral state.

Adherence to the NIV delivery device protocol was supervised daily by a senior physical therapist who completed a checklist that verified whether the interventions were consistently delivered according to the protocol in use. The accuracy of all data collected retrospectively, as well as of the general care protocol noted in the medical record, was guaranteed by a monthly audit of medical records for the purpose of analyzing risk indicators and the quality of hospital care.

An *a priori* sample size estimation was calculated using 80% power, α = 0.05 with F tests as the statistical basis of the calculation using G*Power 3.0 and considering the probability of 32%^(14)^ of being exposed to the nasal mask + binasal prong and developing MDRI stage 1 or 2; thus, a power of 0.8127 was achieved from the calculated total group size of 300 subjects, and 100 subjects in each of the three groups was deemed adequate to determine significant differences between groups.

### Statistical analysis

Data distribution was tested using the KolmogorovSmirnov test. The association between MDRI and possible explanatory variables (sex, length of NIV, NIV mode, type of NIV delivery device) was tested using the chi-squared test. Since the continuous variable presented a distribution that was not normal, comparisons between MDRI scores and birth weight and gestational age were tested by the Mann-Whitney test (nonparametric test) with KruskalWallis *post hoc* analysis.

Bivariate Poisson regression models with robust standard errors were performed to estimate the unadjusted relative risks of presenting no MDRI or with MDRI. Poisson multivariate regression analyses were performed to estimate the relative risks to identify possible predictors associated with the presence of MDRIs. The variables included in the multivariate model were those that were associated with the stages of MDRI in the bivariate analysis considering a p value < 0.20. The relative risks were reported with their respective 95% confidence intervals (95%CIs) and p values = 0.05. All analyses were performed using Statistical Package for the Social Sciences version 20 (SPSS; IBM Corp., Armonk, United States) and presented in the form of tables and graphics.

## RESULTS

A sample of 300 infants was included among the total preterm infants consecutively registered in the neonatal ICU undergoing NIV during the study period. Among these, there were 168 cases without nasal injury with an incidence rate of 56% (95%CI 36.81 - 52), 105 cases with stage 1 with an incidence rate of 35% (95%CI 28.63 - 42.37) and 27 cases of stage 2 with an incidence rate of 9% (95%CI 5.93 - 13.95). Stages 3 and 4 were not found in any group.

The incidence rates according to the nasal septum injury stage and NIV delivery device protocol are shown in table 1. It was observed that there was an injury incidence in stage 1 in all groups, and only switching the NIV delivery device protocol did not present injury in stage 2.

**Table 1:** Incidence of nasal septum injury in infants submitted to noninvasive ventilation with different delivery devices

Device protocol	Stage 1			Stage 2		
	Incidence rate	95%CI	p value	Incidence rate	95%CI	p value
Nasal mask	12.67	8.96 - 17.39		4.00	2.06 - 6.98	
Binasal prongs	11.67	8.13 - 16.23	< 0.001	5.00	2.79 - 8.24	< 0.001
Nasal mask + binasal prongs	10.67	7.30 - 15.06		-	-	

95%CI - 95% confidence interval. p value < 0.01 from the chi-squared test.

The association between NIV delivery devices and nasal injuries is shown in table 1. Significantly fewer skin injuries were found when mask/prongs were systematically rotated when compared to continuous mask or continuous prong groups (n = 68; 40.48%; p value < 0.01), while those who only used binasal prongs presented more stage 2 injuries (n = 15; 55.56%; p value < 0.01).

Table 2 shows the association between the nasal septum injury stages and possible predictor variables.

**Table 2:** Association between demographic, anthropometric, length of noninvasive ventilation and stage of nasal injury

Variables	Without lesion n (%) 168 (56)	Stage 1 n (%) 105 (35)	Stage 2 n (%) 27 (9)		p value
Sex Male	88 (52.39)	53 (50.48)	18 (66.67)		0.31
Female	80 (47.62)	52 (49.53)	9 (33.34)		
Gestational age	35.26 ±3.33^*^	35.03 ± 3.48^*^	32.78 ± 4.31		< 0.01
Birth weight	2747.3 ±807.98^*^	2234.03 ± 693.42	2014.23 ± 1020.62		< 0.01
NIV mode NCPAP	75(44.65)	59(56.2)	10 (37.04)		0.08
NIPPV	93(55.36)	46(43.81)	17 (62.97)		
Length of NIV (days) ≤ 2	50 (29.77)	5 (4.77)	1 (3.71)		
3 - 4	70 (41.67)	15 (14.29)	0 (0)		< 0.01
5 - 6	41 (24.41)	18 (17.15)	2 (7.41)		
> 7	7 (4.17)	67 (63.81)	24 (88.89)		

NIV - noninvasive ventilation; NCPAP - nasal continuous positive airway pressure; NIPPV - nasal intermittent positive airway pressure. Bilevel - bilevel positive airway pressure.

* Different from Stage 2 (p value < 0.01 from Kruskal-Wallis test).

There were no differences between sex and NIV modality. Infants with nasal injury stage 2 had lower gestational age than their counterpart and lower birth weight compared to those without lesions (p value < 0.01). Additionally, staying for more than 7 days on NIV was also associated with a higher frequency of injuries (p < 0.01).

Factors that remained associated with a higher risk of MDRI were birth weight and exposure time to NIV, whereas the type of device was not significant in predicting the chance of nasal injuries in the model presented in table 3. Higher birth weigh was seen as a protective factor against the risk of nasal septum injury, where each gained gram represented a decrease of 1% of the risk of developing nasal septum injury (relative risk - RR: 0.99), while the daily increments of exposure time to NIV increased the risk of MDRI in infants by 4%.

**Table 3:** Adjusted relative risks associated with the incidence of nasal septum injury and associated factors

Variables	Model 1				Model 2			
	RR	95%CI		p value	RR	95%CI		p value
NIV delivery device Nasal mask	1.14	0.90	1.44	0.28	1.11	0.88	1.41	0.37
Binasal prongs	1.14	0.90	1.44	0.28	1.14	0.90	1.44	0.28
Nasal mask + binasal prongs	Ref.	-	-	-	Ref.	-	-	-
NIV mode NCPAP					1.05	0.85	1.29	0.67
NIPPV					Ref.	-	-	-
Gestational age					1.00	0.97	1.03	0.85
Birth weight					0.99	0.99	0.99	0.04^*^
Length of NIV					1.04	1.02	1.06	< 0.01†

RR - relative risk; 95%CI - 95% confidence interval; NIV - noninvasive ventilation; NCPAP - nasal continuous positive airway pressure; NIPPV - nasal intermittent positive airway pressure; Ref. - reference. Statistical significance:

* p < 0.05; †p < 0.01.

## DISCUSSION

In this study, by comparing a protocol using two different NIV delivery devices among three groups (nasal prongs *versus* nasal mask *versus* rotation of nasal prongs and nasal mask) in preterm infants, the rotation every 12 hours of the NIV delivery device protocol was found to be superior to nasal prongs and nasal masks in reducing the incidence of moderate to severe nasal septum injury. In fact, none of the infants in the rotation group had stages 2, 3 or 4 nasal injuries.

There has been increased use of NIV over the last few decades through different modalities for treating acute and chronic respiratory disorders commonly encountered in infants and children.^(23)^ Tight-fitting NIV delivery devices are required to maintain constant airway pressure for effective delivery. However, immaturity and the force applied to the delicate tissues of the nares and nasal septum can compromise skin integrity and cause nasal injury.^(24)^ Reducing morbidity related to using this therapy will enable successful ventilation of this vulnerable population who would otherwise require more invasive therapies.

Only two randomized trials^(11,18)^ evaluated the incidence and severity of nasal injury when rotating two different nasal devices (masks and prongs). These studies reported diverging results regarding the occurrence of injury in association with the NIV delivery device. Bashir et al.^(11)^ showed that the incidence of nasal injury in the mask continue group (33.3%) was significantly lower than that in the prong continue group (91.6%) and rotation group (56.9%) (p < 0.0001). Of note, the studied population was submitted to NIV in approximately 29.5 hours, and the authors do not clarify how long the injury appeared after NIV exposure. In contrast, Newnam et al.^(18)^ demonstrated a reduced incidence of nasal injury (erythema p < 0.001, excoriation p = 0.007) with a rotating nasal mask/prong every four hours compared with single NIV delivery devices.

The present study shows that MDRI secondary to NIV was a very frequent complication in the studied population, with an incidence rate of 44%, regardless of NIV delivery device. These results are comparable to those reported in a systematic review of 45 studies showing an incidence ranging from 20 to 100%.^(21)^ According to the association with protocol and the type of nasal injury, our results shown in the groups undergoing a single NIV delivery device (prongs or mask) presented the same incidence rate of no MDRI (16.67%), stages 1 (11.67% and 12.67%) and 2 (8.96% and 8.13%), respectively.

In contrast, in a cohort study comparing different nasal prongs, Bonfim et al.^(14)^ reported an incidence rate of 52.3% with stage 1, 36.4% with stage 2 and 11.3% with stage 3.

It was observed that staying for more than seven days with NIV was associated with a higher frequency (88.89%) of stage 2 using a single NIV delivery device, even using the ideal prong size following the manufacturer’s recommendations. Different findings were verified by Nascimento et al.,^(25)^ in which the frequency of MDRI (19.7%) with the use of CPAP with nasal prongs appeared from a period of three days. It is worth noting that the authors mentioned that nasal prongs used in all studied newborns were smaller than the ideal size according to weight.^(25)^ Bonfim et al.^(14)^ observed that three to four days of NIV showed a 22.7% frequency for developing stages 2 and 3 using only nasal prongs when compared to the group of infants without injury.

The rotation of the NIV delivery device group of our study showed no cases of stages 2, 3 and 4. Similar to our results, no case of stage 3 disease was reported by Dai et al.^(17)^ in a prospective observational study with a rotating NIV delivery device protocol. The findings of the present study and those of Dai et al.^(17)^ differ from Fischer et al.,^(15)^ who reported that newborns using an alternating NIV delivery device protocol presented a rate of 0.7% of more severe injuries; however, the authors grouped stages 2 and 3 as a single stage, without stratifying the severity. The appearance of injuries was reported by Fischer et al.^(15)^ after 26 days of NIV use. Similarly, Xie et al.^(16)^ observed MDRI stage 3 after three weeks under NIV using an alternating NIV delivery device protocol when nasal hyperemia appeared.

Such facts may suggest that rotating devices during NIV, as well choosing the ideal size following the manufacturer’s recommendations, may delay the appearance of nasal injury and should be used to establish an NIV delivery device protocol. Moreover, we believe that changing the type of NIV delivery device should be performed not only whenever erythema is noticed but also systematically for no more than 12 hours. This would reduce pressure on specific points and consequently the risk of MDRI.

No association was found between the type of NIV delivery device and gestational age. However, higher birth weight is predicted to be protective, and the addition of days using NIV increases the risk of MDRI.

Although 1% seems little, each gram of extra birth weight represented a 1% increase in protection from developing nasal septum injury, which seems to be important in clinical practice because between an infant weighing 1000g and another weighing 1025g, the infant with greater weight has 25% more protection against developing injury than the infant weighing 1000g.

Dai et al.^(17)^ found that factors increasing the likelihood of injury were gestational age less than 32 weeks and treatment for more than 6 days with NIV. Fischer et al.^(15)^ reported that newborns weighing less than 1,500g and those staying in the neonatal ICU for more than 14 days were more likely to develop MDRI. Despite not looking for the main outcome of the association of birth weight with the appearance of moderate injury, Bonfim et al.^(14)^ found a greater tendency for more severe injuries in lower birth weight (1000 - 1500g). These data highlight the importance of maintaining or gaining weight in infants, since it appears to provide resistance to the nasal epithelium against the pressures imposed by the devices during NIV therapy.

To the best of our knowledge, only two studies^(11,18)^ in neonatology have evaluated NIV protocols using systematically rotated mask/prongs to prevent adverse events, and the authors presented divergent outcomes. Unlike the results from Newnam et al.^(18)^ and our study, a randomized controlled trial enrolling 175 preterm infants with gestation less than or equal to 30 weeks and respiratory distress supported by NCPAP reported that the incidence of nasal injury in the mask continue group (n = 19/57; 33.3%) was significantly less compared to a prong continue group (n = 55/60; 91.6%) and rotation group (n = 33/58; 56.9%; p value < 0.0001), but the authors did not report how long after NIV exposure the injury appeared; this is possibly because the population in this study spent less than 48 hours on NIV, unlike our study, where participants spent up to 7 days on NIV.^(11)^

The present study addressed a low-cost protocol to prevent MDRI during NIV by using alternating prongs and masks every 12 hours in the neonatal ICU.

This study has some recognized limitations. It was a retrospective study in a single center and employed a convenience sampling method. However, with the retrospective cohort design, it was possible to identify from which day of exposure to NIV the lesion started. Despite the limitation of cohort studies in not revealing cause/effect, they are useful in identifying risk and prognostic factors, in monitoring the natural history of certain diseases, and in studying the impact of diagnostic and therapeutic interventions. Furthermore, as previously mentioned, the required sample size (300 infants) to detect differences between NIV delivery device protocols at a power of 0.8127 was achieved.

Although we did not adopt a pressure injury risk assessment scale, checking the MDRI is part of the collaborative practice between nurses and physical therapists in the routine of our neonatal ICU. As the senior physiotherapist performed a daily checklist on the full execution of the protocol and the correct filling of the patient’s electronic medical record, we believe that our data are consistent with the study methodology and reveal the current practice.

Future well-designed clinical trials are required to reinforce the recommendation of rotating devices in NIV protocols associated with general care protocol for handling NIV delivery device as an important strategy to prevent MDRI.

## CONCLUSION

In conclusion, no incidence of medical device-related injuries at stages 2, 3 or 4 was found in preterm infants in the current cohort study due to rotating nasal prongs and masks during noninvasive ventilation therapy. Noninvasive ventilation delivery with a single device predisposes infants to a greater frequency of medical device-related injuries at stage 2. An increased risk of injuries was shown when preterm infants used noninvasive ventilation for more than 7 days. Larger birth weight preterm infants had less nasal septal injury regardless of the type of noninvasive ventilation delivery device.
